# Gearbox Fault Diagnosis Method Based on Multidomain Information Fusion

**DOI:** 10.3390/s23104921

**Published:** 2023-05-19

**Authors:** Fengyun Xie, Gan Wang, Jiandong Shang, Hui Liu, Qian Xiao, Sanmao Xie

**Affiliations:** 1 School of Mechanical Electrical and Vehicle Engineering, East China Jiaotong University, Nanchang 330013, China; 2 State Key Laboratory of Performance Monitoring Protecting of Rail Transit Infrastructure, East China Jiaotong University, Nanchang 330013, China; 3 Life-Cycle Technology Innovation Center of Intelligent Transportation Equipment, Nanchang 330013, China

**Keywords:** gearbox, singular value decomposition, convolutional neural network, support vector machine, fault diagnosis

## Abstract

Traditional methods of gearbox fault diagnosis rely heavily on manual experience. To address this problem, our study proposes a gearbox fault diagnosis method based on multidomain information fusion. An experimental platform consisting of a JZQ250 fixed-axis gearbox was built. An acceleration sensor was used to obtain the vibration signal of the gearbox. Singular value decomposition (SVD) was used to preprocess the signal in order to reduce noise, and the processed vibration signal was subjected to short-time Fourier transform to obtain a two-dimensional time–frequency map. A multidomain information fusion convolutional neural network (CNN) model was constructed. Channel 1 was a one-dimensional convolutional neural network (1DCNN) model that input a one-dimensional vibration signal, and channel 2 was a two-dimensional convolutional neural network (2DCNN) model that input short-time Fourier transform (STFT) time–frequency images. The feature vectors extracted using the two channels were then fused into feature vectors for input into the classification model. Finally, support vector machines (SVM) were used to identify and classify the fault types. The model training performance used multiple methods: training set, verification set, loss curve, accuracy curve and t-SNE visualization (t-SNE). Through experimental verification, the method proposed in this paper was compared with FFT-2DCNN, 1DCNN-SVM and 2DCNN-SVM in terms of gearbox fault recognition performance. The model proposed in this paper had the highest fault recognition accuracy (98.08%).

## 1. Introduction

Gearboxes are mainly composed of gears, shafts, bearings and casings. With the continuous development of modern science and technology, their application is becoming ever more extensive [1]. They are currently used in mechanical transmission systems such as aero-engines, wind power, petrochemicals and metallurgy [2,3]. The working environment of the gearbox is usually very harsh. Under high-speed and heavy-load operating conditions, the internal parts of a gearbox are easily damaged and can even stop working [4,5], leading to economic losses and casualties. Therefore, fault diagnosis has great significance for gearboxes [6].

A large number of scholars have carried out research on gearbox fault diagnosis. Liu et al. [7] used a combination of empirical mode decomposition and the Hilbert spectrum for gearbox fault diagnosis. Cheng et al. [8] proposed a fault diagnosis method using singular value and empirical mode decomposition to extract the characteristics of gear- and roller-bearing vibration signals, and then used support vector machines for pattern recognition and classification. Wang et al. [9] proposed a gearbox fault diagnosis method based on a combination of recursive graphs and 2DCNN. First of all, the vibration signal was converted into a recursive graph, and this was then input into the 2DCNN model for gearbox fault pattern recognition and classification. Chen et al. [10] proposed a gearbox fault diagnosis method based on CNN–SVM. This study used a CNN model for feature extraction, and then the researchers input the feature information into SVM for pattern recognition and classification and achieved good results. There are also many experts who analyzed gear failures and conducted experimental verification in the form of dynamic modeling [11,12]. They conducted their research by discussing how response curves and spectra related to actual failure modes [13,14]. With the continuous development of the field of computer science and gradual expansion of its scope of application, fault diagnosis technology has also begun to develop from traditional fault diagnosis into intelligent fault diagnosis [15].

Traditional fault diagnosis is mainly divided into two aspects: feature extraction and pattern recognition [16]. Feature extraction and selection are mainly manual. The design of the shallow structure of the classifier itself has limitations when dealing with large-scale data and is difficult to adapt to the intelligent development trend of fault diagnosis [17]. Therefore, there is great interest from researchers in developing methods to improve the accuracy, intelligence, versatility and practical application ability in gearbox fault diagnosis engineering, and realize intelligent gearbox diagnosis based on big data [18,19].

In recent years, against the background of artificial intelligence, there has been continuous innovation and development in deep learning technology [20]. This technology is widely used by scholars at home and abroad in the fields of automatic driving, big data processing, image recognition, etc. [21,22]. The most important aspect of deep learning is its feature learning ability, which can adaptively learn the mapping relationship between input and output in data, and its capacity to mine the nonlinear information hidden in the deep layers of data [23]. Deep learning technology has natural advantages in the intelligent diagnosis of gearboxes. It can unify the two aspects of feature extraction and pattern recognition [24] and realize the entire process of end-to-end fault diagnosis without manual intervention. As a consequence, scholars are increasingly using convolutional neural network (CNN) [25], dynamic Bayesian network (DBN) [26], recurrent neural network (RNN) [27] and other deep learning methods to study intelligent fault diagnosis methods for application to gearboxes based on characteristics of the industrial scenarios in which gearboxes are operated.

Although the application of deep learning in fault diagnosis has achieved some initial results, most of the research has been based on one-dimensional vibration signal data as the input. This method has a lot of feature information and is complicated and difficult to screen. It has problems such as incomplete feature information, heavy extraction workload and easy signal interference. To a certain extent, it limits the further improvement of gearbox fault diagnosis accuracy and the optimization of diagnosis efficiency [28].

Deep learning techniques have a natural advantage in processing images. It is therefore worth exploring the potential advantages of a bearing fault diagnosis method that converts the vibration signal into two-dimensional image data and then uses the two-dimensional image data as the input into the deep learning model [29]. Visualizing the vibration signal can not only capture high-quality information from the vibration signal, but also optimize the preprocessing of the vibration signal data. In addition, the deep learning model has good recognition and processing characteristics in relation to the converted two-dimensional image data [30].

CNN is a typical deep learning model. Currently, 1DCNN and 2DCNN are commonly used. The model extracts the signal features layer by layer through convolution, pooling and nonlinear activation function mapping [31]. Compared with the fully connected deep learning model, CNN is more robust and has better generalization ability. It can improve network performance and reduce training costs through weight setting and pooling [32], and the overfitting phenomenon is uncommon when using it.

In order to effectively improve the complexity and incompleteness of manual feature extraction in the fault diagnosis process of railway vehicle gearboxes, the workload of the extraction should be high, the signal should be susceptible to noise interference, the interference caused by the impact of external experimental environment noise on fault recognition should be reduced, and more complete feature information should be saved. This article proposes a gearbox fault diagnosis method based on multidomain information fusion CNN. The contributions of this paper are as follows:

(1) An experimental platform for gearbox fault diagnosis was built, and a gearbox fault diagnosis method based on multidomain information fusion CNN was proposed. The method was verified as having high robustness and feasibility.
(2) The SVD algorithm was used to preprocess and denoise the original signal of the gearbox. In terms of SVD signal reconstruction, a singular value energy difference spectrum was introduced. This method determines the effective order of the reconstruction matrix after singular value decomposition based on the contribution of noise signals and useful signals to singular values.
(3) The one-dimensional gearbox vibration signal and the two-dimensional frequency map of STFT time and CNN were combined. CNN multifeature fusion was used to enrich the features of the two different dimensions, which reduced the problem of gearbox information loss during the adaptive extraction process.

This paper is composed as follows: Section 2 introduces the relevant algorithm principles used: SVD, STFT, 1 DCNN, 2 DCNN and SVM; Section 3 describes the construction of the relevant fault diagnosis models; Section 4 covers the building of the gearbox fault diagnosis experimental platform and the data collection; Section 5 sets out the experimental analysis and verification; and Section 6 presents the conclusions of this study.

## 2. Principle Introduction

### 2.1. SVD

The singular value decomposition (SVD) method is currently used in data dimensionality reduction, image processing, signal processing and other fields [33]. In the field of signal processing, SVD has been successfully used for signal noise reduction. In the practical application of SVD, the determination of the effective order of the reconstructed matrix after decomposition is a challenge. Some scholars propose the use of the singular entropy increment and threshold method to select the reconstruction order, but these methods often rely on user experience, and the subsequent noise reduction effect is not ideal. In order to solve this problem, this paper introduces the singular value energy difference spectrum [34] and determines the reconstruction order according to the energy contribution of the signal and noise to the singular value, thereby achieving the noise reduction of the vibration signal. The main principles of SVD are as follows:

For the gearbox vibration signal, $X=\{x_{1},x_{2},x_{3},\cdots ,x_{N}\}$ because the vibration signal is usually a one-dimensional signal. SVD cannot be directly performed on it, and a two-dimensional matrix needs to be constructed first. There are many ways to construct a two-dimensional matrix from a one-dimensional signal, such as via the circular, Toeplitz and Hankel matrices. The Hankel matrix is the most widely used because of its zero-phase-shift characteristics and wavelet-like characteristics, and so we first construct the Hankel matrix $A_{m\times n}$ for X:

(1) $$A_{m\times n}=\left[\begin{array}{ccc} x(1) & \cdots & x(n)\\ \cdots & & \cdots \\ x(m) & \cdots & x(N) \end{array}\right]=D_{m\times n}+W_{m\times n}$$

where $A_{m\times n}$ is a Hankel matrix constructed for pairs, N=m+n+1, $D_{m\times n}$ is useful signal space and $W_{m\times n}$ is noise signal space. When m=N/2, the Hankel matrix has a good noise reduction effect.

In terms of signal reconstruction, the determination of the useful order of singular values is particularly important. If more singular values are selected for signal reconstruction, a part of the noise signal remains in the signal after noise reduction, and the noise reduction will be incomplete. However, if fewer singular values are selected for signal reconstruction, useful signals will be deleted, resulting in incomplete information in the original vibration signal [35]. In this paper, the singular value energy difference spectrum is introduced, and the effective order of the reconstruction matrix after singular value decomposition is determined according to the contribution of the noise signal and the useful signal to the singular value. The signal energy is shown in Formula (2):

(2) $$E=\sum_{i=1}^{q}\sigma_{i}^{2}$$

In Formula (2), E represents the signal energy. $\sigma_{1}$,$\sigma_{2}$, …,$\sigma_{k}$ are the singular values of the matrix $A_{m\times n}$, and q represents the total order, that is, up to q. Therefore, the singular value energy difference spectrum is defined and normalized:

(3) $$p(i)=\frac{\sigma_{i}^{2}-\sigma_{i+1}^{2}}{E}$$

Here, the sequence formed by p(i)(i=1,2,⋯,q) is called the singular energy difference spectrum, and Formula (3) represents the energy change represented by adjacent singular values. The singular value energy of the useful signal accounts for a larger proportion than the noise signal, meaning that it will cause greater peak fluctuations at the boundary between the noise signal and the useful signal. The singular value after the peak is mainly generated by the noise signal, meaning that the singular value corresponding to this point can be found in the singular value energy difference spectrum. We then take this point as the order of the reconstructed signal, which enables the separation of noise signal and useful signal and succeeds in reducing the noise of the gearbox vibration signal.

### 2.2. STFT

The short-time Fourier transform (STFT) is also referred to as a windowed Fourier transform. Because the Fourier transform is only suitable for steady-state signal analysis, and as unsteady-state signals are very common in mechanical equipment, the short-time Fourier transform is a method developed to adapt to unsteady-state signal analysis [36]. This method can transform the one-dimensional gearbox vibration signal into a two-dimensional matrix containing feature information in the time–frequency domain, which can then be input into the 2DCNN. The main principle is to process a non-stationary signal with a square frame, where the time inside the frame is regarded as a stationary signal. The square frame here is equivalent to a window, and so it is also called windowing. The window function is multiplied with the signal and then the Fourier transform is performed to obtain the spectrum information. A series of spectrum information is obtained by moving the window function, and splicing these together produces data with a frequency that changes with time [37]. The short-time Fourier transform expression is as follows:

(4) $$X(\omega ,\tau )=\int_{-\infty }^{\infty }x(t)w(t-\tau )e^{-j\omega t}dt$$

**Figure 1:**
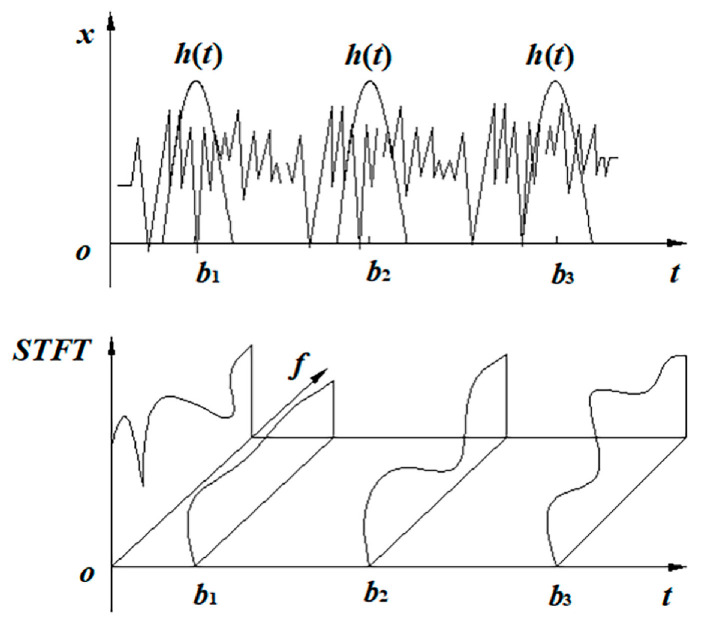
Schematic diagram of STFT.

In Formula (4), ω represents the frequency, τ represents the starting time of the current window and X(ω,τ) represents the contribution of the signal component with frequency ω in the window at time τ. t is the period of time, x(t) is the unsteady signal and w(t−τ) is the window function. The schematic diagram of STFT is shown in Figure 1.

Assume that h(t) in Figure 1 is a window function, and that $b_{1}$, $b_{2}$, and $b_{3}$ are time periods. When h(t)=1, the short-time Fourier transform is restored to the Fourier transform. The choice of window function and window width are important factors affecting the effect of STFT. A suitable window function can effectively reduce the spectrum leakage caused by the interception of the original nonstationary signal. The selection of window width will affect the resolution in the time and frequency domains. If the window is too narrow, the signal in the window will be too short and the accuracy of frequency domain resolution will not be high. If the window is too wide, the time domain will not be sufficiently fine and the time resolution will be low.

### 2.3. CNN

Convolutional neural network (CNN) is a feedforward neural network that has become one of the most commonly used algorithms in the field of deep learning in recent years, particularly in the field of pattern classification [38]. The network can avoid image preprocessing in the early stage, and the original image can be directly input. A typical convolutional neural network mainly consists of an input layer, a convolutional layer, a pooling layer, a fully connected layer and an output layer.

(1) Input layer. The CNN input layer can preprocess the input data, such as standardization, normalization, etc.
(2) Convolutional layer. The convolutional layer is the core component of CNN. Its largest feature is weight sharing, which can be realized through the convolution kernel. The convolutional layer uses the convolution kernel to locally operate on the input data to extract the corresponding features of this part. As the number of convolutional layers deepens, the required parameters also increase. Deeper features can also be extracted. The convolution operation expression is as follows:
  (5) $$x_{j}^{l}=f(\sum_{i\in m_{j}}x_{i}^{l-1}\cdot k_{ij}^{l}+b_{j}^{l})$$
  In Formula (5), the number of convolutional layers is l, the output of the l layer is $x_{j}^{l}$, the input of the l layer is $x_{i}^{l-1}$, the weight matrix is $k_{ij}^{l}$, the bias is $b_{j}^{l}$, the activation function used by the convolutional layer is f(⋅) and $m_{j}$ is the jth convolution area of the feature map (l−1) layer.
(3) Pooling layer. Pooling layers are also called downsampling layers. This layer mainly performs feature extraction and dimensionality reduction during the running of the CNN, which can reduce the amount of calculation required. To a certain extent, it can also reduce the possibility of overfitting. The maximum pooling formula is:
  (6) $$P^{l(i,t)}=\underset{(j-1)\omega +1\leq t\leq j\omega }{max}\{a^{l(i,t)}\}$$
  In Formula (6), $P^{l(i,t)}$ means maximum pooling, the number of pooling layers is l, t represents the activation value, ω represents the pooling width, the minimum value of t is (j−1)ω+1 and the maximum value range is jω. The t-th activation value of the i-th eigenvalue of the l layer is $a^{l(i,t)}$.
(4) Fully connected layer and output layer. After the previous convolutional and pooling rounds, the fully connected layer of the image input is fully connected between the input and output. This mainly summarizes the features extracted by the convolutional layer and the pooling layer to achieve global optimization [39]. The Softmax function is generally used as the classifier of the output layer. However, as the Softmax classifier leads to insufficient generalization ability of the graphical model and is not suitable for image classification, here we instead use SVM.

2DCNN is widely used in the field of image recognition and is effective for image feature extraction and classification [40]. It differs from 1DCNN in the dimensions of the input data. The input of 2DCNN comprises two-dimensional or three-dimensional data. In our study, the two-dimensional time–frequency image generated by the short-time Fourier transform is input into the 2DCNN.

### 2.4. SVM

Support vector machine (SVM) is a data analysis method developed on the basis of statistics. Its basic principle is to map the nonlinear problem in the original low-dimensional input space onto the high-dimensional feature space for solution, and it is often used in classification and regression analysis and to solve other problems [41].

In SVM nonlinear data classification, the input data are mapped onto the high-dimensional space primarily via the kernel function. The selection of different kernel functions has an impact on the classification effect. Kernel functions include polynomial, Laplacian and radial basis function kernels. We selected the radial basis function kernel for our study because it has a wider range of applications. The main principle of SVM is shown in Formula (7):

(7) $$\left\{\begin{array}{c} min\frac{1}{2}∥w{∥}^{2}+C\sum_{i=1}^{m}\xi_{i}\\ s.t.y_{i}\left(\left(w\cdot x_{i}\right)-b\right)³\geq 1-\xi_{i}\\ i=1,2,\cdots ,m,\xi_{i}\geq 0 \end{array}\right.$$

In Formula (7), the given data set is $\left\{(x_{1},y_{1}),(x_{2},y_{2}),\cdots ,(x_{m},y_{m})\right\}$. The input feature vector is $x_{i}$, which for the label is $y_{i}\in \{-1,1\}$. Classification samples are i=1,2,⋅⋅⋅,m, the weight is w, the penalty factor is C, and w and b are the parameters to be optimized. $\xi_{i}$ is the relaxation component of the *i*-th component, linearly separable when $\xi_{i}$ = 0. The hyperplane used for classification is obtained using the above formula. Then, the appropriate kernel function and parameters are selected, and the classification discriminant function is used to judge the category of x.

## 3. Fault Diagnosis Model Construction

### 3.1. 1DCNN + 2DCNN + SVM Model

**Figure 2:**
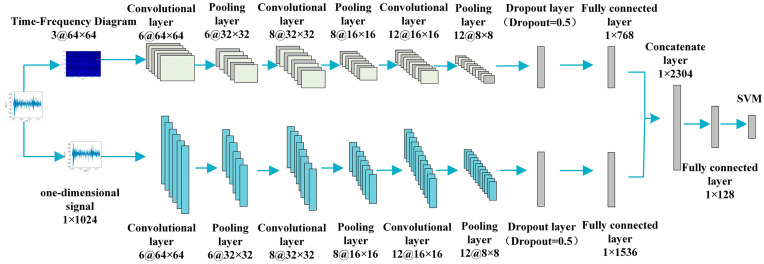
1DCNN + 2DCNN + SVM workflow diagram.

The overall workflow of the 1DCNN + 2DCNN + SVM model is shown in Figure 2. The upper channel 2DCNN and the lower channel 1DCNN work simultaneously. The feature vectors extracted via the two channels are then combined into one fused feature vector. Finally, SVM is used to identify and classify the fault types. The multidomain information fusion CNN structure parameters are shown in Table 1.

Table 1 sets out the multidomain information fusion CNN model network structure parameters. The training parameters were set as follows. According to the setting of the sample label, this was divided into 4 categories, meaning that the number of nodes in the final output layer was 4. The size of the time–frequency map in the upper channel 2DCNN was 64 × 64, and the fault features were extracted after three rounds of convolution and pooling. The first layer of convolution had 6 convolution kernels, the second has 8 and the third has 12. In the lower-channel 1DCNN model, the sample length of the input gearbox vibration signal was 1024. Again, after three rounds of convolution and three of pooling to extract fault diagnosis, the number of convolution kernels in the first, second and third layers was the same as above. Of these, batch size = 64, learning rate = 0.001 and the activation function of each layer used ReLU. Pooling was max pooling, and padding was set to “same”. We selected the Adam algorithm for optimization. In order to prevent the model from overfitting, a dropout layer was added after the expansion layer: dropout = 0.5. The sample feature set output by the fully connected layer was used as the input sample of the SVM model for final classification purposes.

### 3.2. Multidomain Information Fusion Model

**Figure 3:**
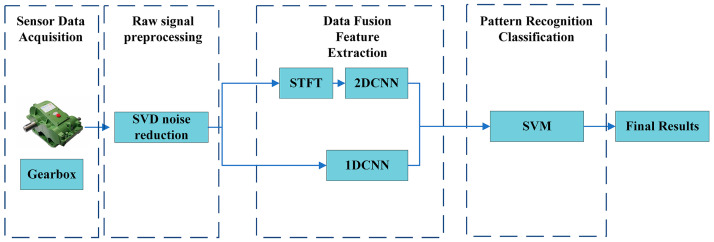
Flow chart of multidomain information fusion model.

The significance of multidomain information fusion lies in the integration of several excellent models via scientific methods for the purpose of removing the bottleneck of the generalization ability of a single model of unknown problems. Furthermore, the advantages of multiple models can be combined to achieve the optimal solution to a problem [42]. The model fusion in this paper is mainly divided into four parts: sensor data acquisition, vibration signal preprocessing, data fusion feature extraction and pattern recognition classification. The overall flowchart of the fault diagnosis model is shown in Figure 3.

As shown in Figure 3, the first part is sensor data acquisition. The acceleration sensor acquires the vibration signals of the four gearbox states: pitting, broken teeth, wear and normal. The second part is the preprocessing of the original gearbox vibration signal. In this study, SVD is used for noise reduction processing. The third part is feature extraction. The noise-reduced vibration signal is subjected to dual-channel CNN simultaneous feature extraction. The upper channel performs STFT transformation on the signal data to obtain a two-dimensional spectrum image, and then places the two-dimensional spectrum image into the constructed 2DCNN network model. The lower channel places the data into the constructed 1DCNN network model. Finally, in pattern recognition and classification, SVM is used for classification to obtain the final fault diagnosis result.

## 4. Fault Diagnosis Experimental Setup and Data Collection

**Figure 4:**
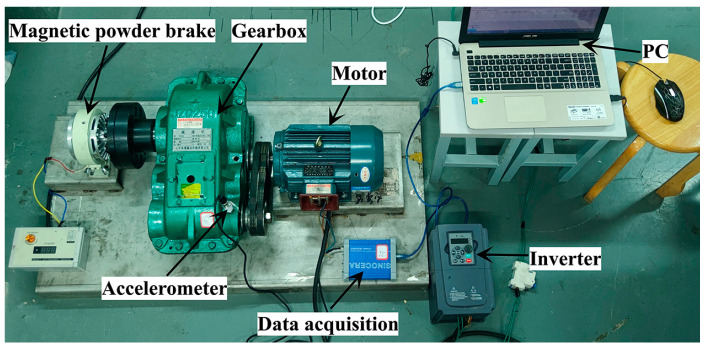
Gearbox fault diagnosis experimental platform.

In order to verify the actual effect of the method proposed in this paper in gearbox fault diagnosis, this experiment used the JZQ250 fixed-axis gearbox for fault diagnosis research. The experimental platform is shown in Figure 4.

**Figure 5:**
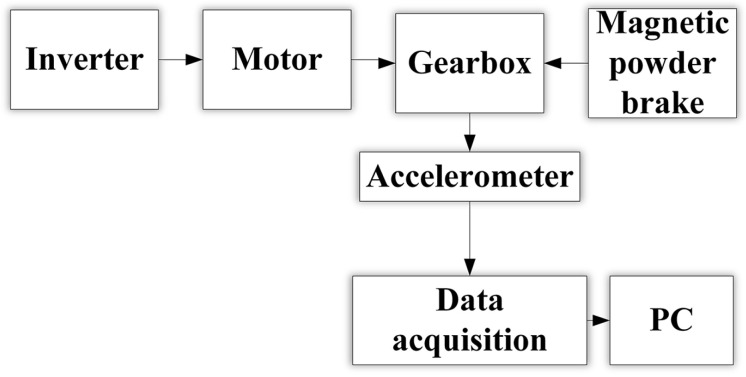
Gearbox fault diagnosis experiment flow chart.

It can be seen from Figure 4 and Figure 5 that the platform was mainly composed of a PC, a data acquisition card (model YE6231), a piezoelectric acceleration sensor (model CAYD051V), a gearbox, a magnetic powder brake, a three-phase asynchronous motor (model YE2-100L2-4) and an inverter (model G7R5/P011-T4). The specific operation steps were as follows:

(1) An air switch was added between the inverter and the power plug to ensure that the experimental process was carried out under safe conditions;
(2) The motor was connected to the frequency converter, and then the gearbox and the motor were connected by a belt. The magnetic powder brake and the gearbox were connected via coupling.
(3) A piezoelectric acceleration sensor was installed at the axial position of the high-speed shaft end cover of the gearbox and was connected to a PC via an acquisition card.

This was a no-load gearbox experiment, meaning that the magnetic powder brake was closed. In terms of fault diagnosis experiment design, system variability and limited fault coverage will affect the accuracy and reliability of fault diagnosis techniques. As most internal failures in gearboxes occur in the gears, we primarily focused on the gears. The type of gear measured in the experiment is shown in Figure 3, the motor speed was 900 r/min, and the frequency was 6 kHz. The specific data are shown in Table 2.

It can be seen from Table 2 that the gearbox fault diagnosis experiment was divided into four states: pitting, broken teeth, wear and normal. The length of each group of data was 1024 points.

The number of training, verification and test sets are shown in Table 3.

As can be seen from Table 3, this study used a total of 4000 sets of sample data. These consisted of 1000 sets of pitted gears, 1000 sets of broken teeth, 1000 sets of worn gears and 1000 sets of normal gears. The corresponding labels were 0, 1, 2, and 3, and these were divided into 2800 sets of training sets, 800 sets of verification sets and 400 sets of test sets.

## 5. Experimental Analysis and Verification

### 5.1. Gearbox Vibration Signal Preprocessing

**Figure 6:**
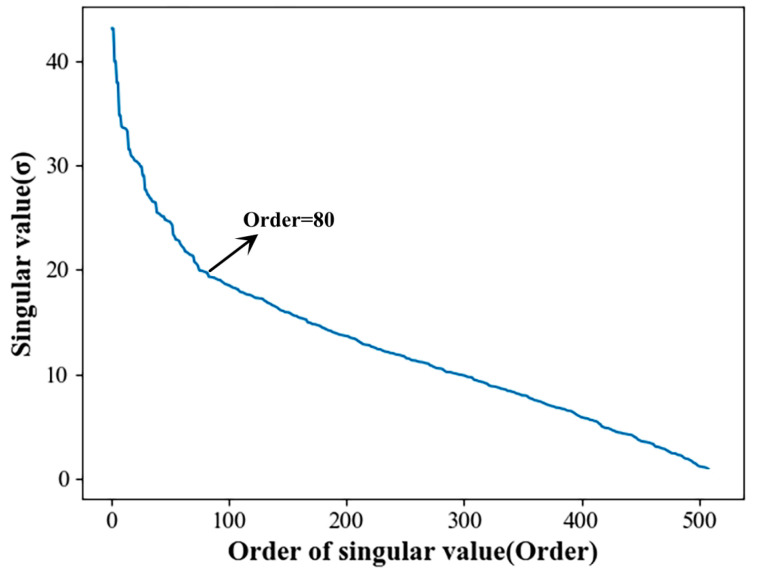
Singular value distribution curve of the original noisy signal.

**Figure 7:**
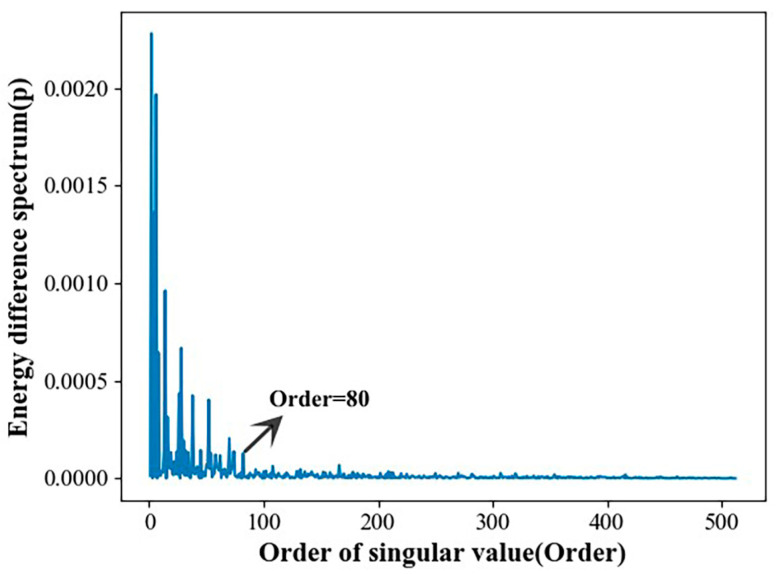
Singular value energy difference spectrum curve of the original noisy signal.

**Figure 8:**
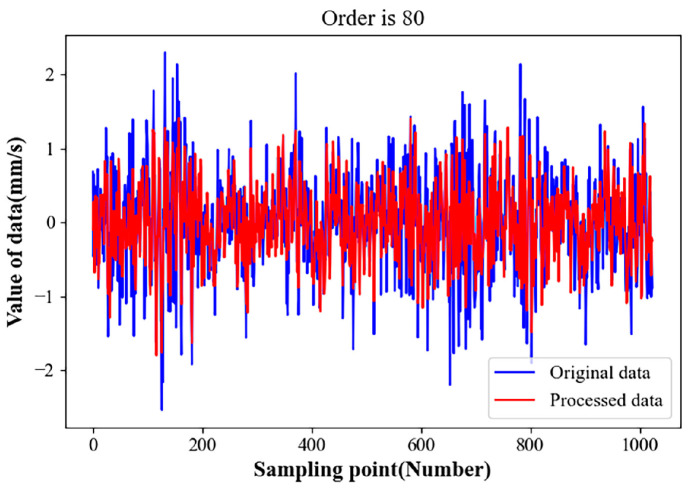
Comparison of vibration signals before and after noise reduction.

The collection of original vibration signals through gearbox fault diagnosis experimental platforms is usually accompanied by a lot of noise and the aliasing of multiple frequency components, meaning that filtering noise signals and effective feature extraction are very important steps [43]. For this reason, singular value decomposition of the noise-containing signal was carried out in this study. Figure 6 shows the singular value distribution curve of the original noisy gearbox vibration signal. Figure 7 shows the singular value energy differential spectrum curve of the original noise-containing signal calculated according to Formula (3). To increase the convenience of observation, the first 500 singular values were taken for analysis in this example. Figure 8 is a comparison of the vibration signal of the gearbox before and after noise reduction.

It can be seen from Figure 6 that the noise signal is located after the order of singular value 100, and that the singular value is relatively small and gentle. The useful signal is located before singular value order 100, whereas the singular value is larger. The peak signal in Figure 7 corresponds to the position where the singular value in Figure 6 changes abruptly. It can be seen that, when the singular value order is 80, the peak signal in Figure 7 becomes flat. According to the definition of the energy difference spectrum, this indicates the boundary point between the useful signal and the noise signal. Therefore, the reconstruction order was taken to be 80; that is to say, the first 80 singular values were taken for signal reconstruction, and the later singular values were taken as zero. The signal comparison before and after noise reduction is shown in Figure 8. It can be seen that, after the noise reduction processing of the singular value energy difference spectrum, the signal mutation partly becomes smooth. The overall periodicity of the signal is more obvious, and the noise signal is basically well suppressed. This shows that it is feasible to determine the order of the reconstructed signal based on the singular value energy difference spectrum.

### 5.2. Time–Frequency Map Obtained by STFT

**Figure 9:**
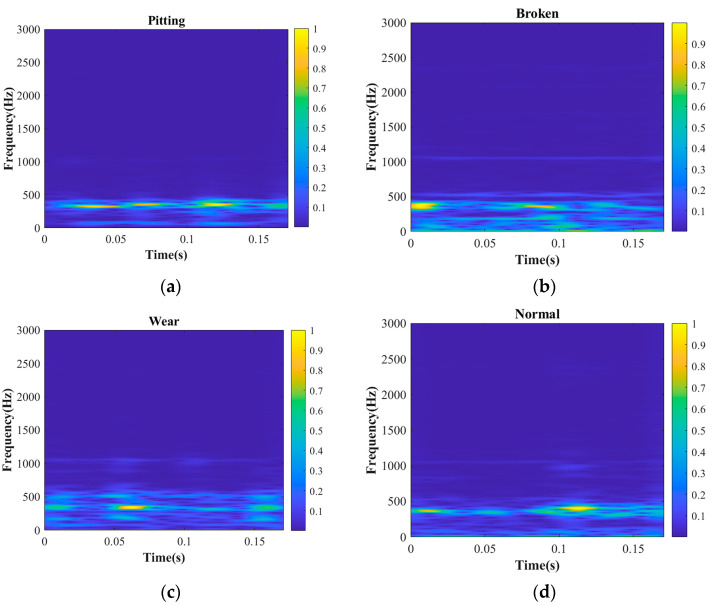
Time–frequency diagram of gearbox in different states: (**a**) pitting; (**b**) broken; (**c**) wear; and (**d**) normal.

STFT was performed on the preprocessed gearbox vibration signal to obtain a two-dimensional time–frequency diagram. Time–frequency diagrams of the randomly intercepted parts of the gearbox signal data in the four states are shown in Figure 9a–d.

Figure 9 shows the time–frequency diagrams of the gearbox under the four states of pitting, broken teeth, wear and normal. The time–frequency analysis method simultaneously presents the time, frequency and energy (amplitude) of the gearbox signal in the form of a time–frequency diagram. This is also a popular method for dealing with nonstationary signals [44]. The time–frequency image obtained using STFT has good time–frequency resolution and can accurately express gearbox vibration signal data. In this study, STFT was used to analyze the time–frequency of the gearbox vibration signal, and the STFT time–frequency diagram was obtained. Then, 2DCNN was used to extract the time–frequency map information for feature extraction.

### 5.3. Overall Model Analysis of Fault Diagnosis

**Figure 10:**
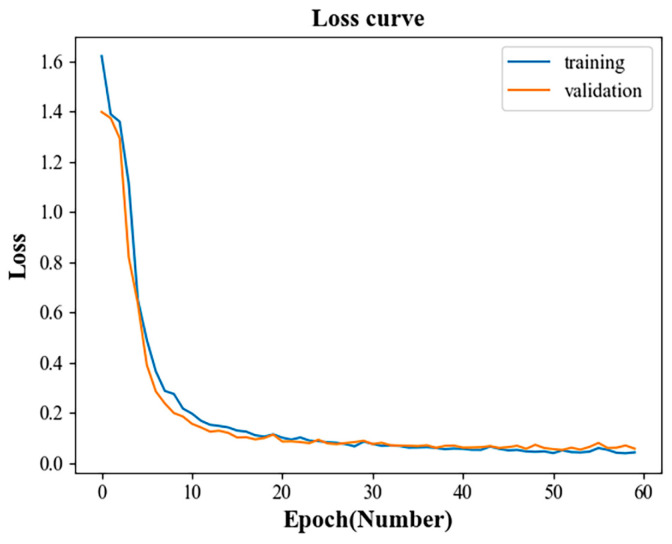
Loss value change curves of training and verification sets.

**Figure 11:**
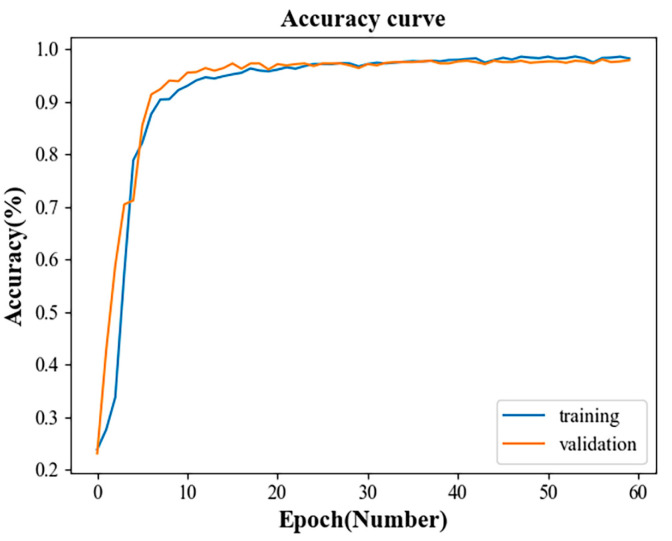
Accuracy change curve of the training and validation sets.

The computer operating system used in this experiment was Windows 11, the programming language was python3.7 and the deep learning framework was Keras. In order to verify whether the fault accuracy of the multidomain information fusion CNN network model proposed in this paper was as expected, the entire data set was divided into training, verification and test sets. The training and verification set were first used to train the multidomain information fusion CNN network model. Then, the test set was input into the trained model and the result was output to obtain the accuracy of the test set. Figure 10 and Figure 11 show the change curves of the loss value of the model training and verification sets and the accuracy rate change curves of the training and verification sets.

From the loss value change curves of the training and verification sets in Figure 10, it can be seen that the loss value of the training sample and the verification sample decreased continuously with the increase in epoch until finally tending to be relatively stable. In the first 10 iterations, the loss values of the training and verification samples dropped very rapidly, and the rates of decline of the two basic curves continued to change synchronously. The loss value dropped from around 1.6 to around 0.2, which showed that the model was converging rapidly. Within 10 to 30 iterations, the rate of descent of the training and validation samples slowed down significantly compared to the first 10 iterations. The loss value dropped from about 0.2 to about 0.1, which indicated that the model was still learning and had a tendency to converge. After 30 iterations, the loss values of the training and verification samples gradually approached 0. The two curves also basically overlapped, and there was no further change in the loss value. This indicated that the model had completed training and had good convergence.

From the change curve of the accuracy rate of the training and verification sets shown in Figure 11, it can be seen that, within the first 10 iterations, the accuracy of the training sample and the verification sample rose rapidly and fluctuated significantly, rising from about 20% to about 88%. Between 10 and 30 iterations, the accuracy of the training and validation samples increased relatively steadily and slowly, with the accuracy rate increasing from about 88% to about 97%. After 30 iterations, the accuracies of the training and the verification samples were infinitely close to each other and basically remained unchanged. The overall curve was relatively smooth, and there was no large fold line fluctuation. This indicated that the network model had been trained and also proved that the multidomain information fusion CNN network model had fast convergence and high accuracy of fault diagnosis and classification.

### 5.4. t-SNE Visualization Algorithm and Analysis

**Figure 12:**
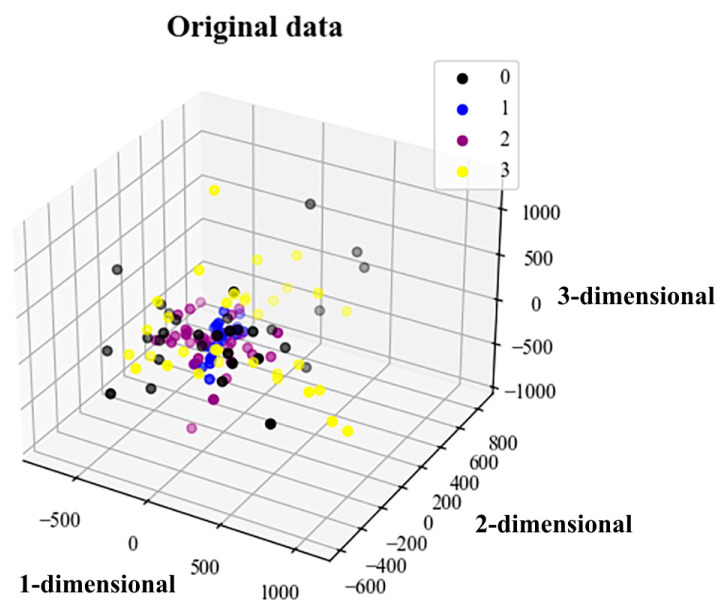
3D plot of original data visualization results.

**Figure 13:**
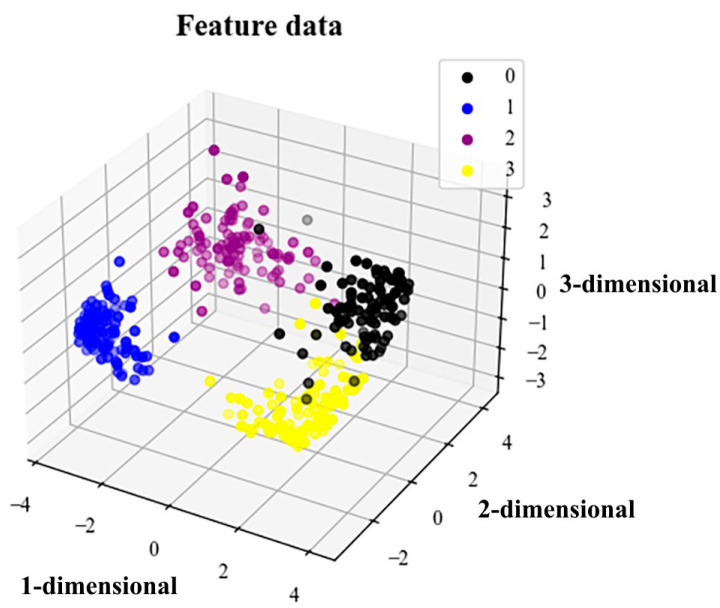
3D graph of data visualization results after training.

t-SNE (t-distributed stochastic neighbor embedding) is a dimensionality reduction technique. It is applied to represent high-dimensional data in a two-dimensional or three-dimensional low-dimensional space and can be used for the visualization of high-dimensional data [45]. This technique was used to visualize the features extracted by the model. Figure 12 and Figure 13 show the original and characteristic data, respectively.

It can be seen from Figure 12 that the initial data distribution is relatively concentrated and disorderly, with labels 0, 1, 2 and 3 each being randomly distributed. After the training of the multidomain information fusion CNN network model proposed in this paper, this can more intuitively reflect the consistency of various types of fault recognition. Figure 13 shows that the four labels of 0, 1, 2 and 3 already have good clustering. The distance between the fault state features of the same label data is small, and the discrimination between label data is obviously increased. This again verifies that the model proposed in this paper has high precision, strong feature extraction ability and good robustness.

### 5.5. Result Analysis

For the final classification, we used the sample feature set output from the fully connected layer (FC layer) as the input sample of the SVM model. The SVM algorithm is essentially a binary classification algorithm, and there are two methods for solving classification problems. The first method is to directly modify the objective function and achieve multiclassification by solving optimization problems. However, this method has high computational complexity and is generally only suitable for use on small sample data. The second method involves combining multiple binary classifiers to construct multiple classifiers, commonly known as the one-to-one method and the one-to-many method. Our study adopted a one-to-one classification method.

**Figure 14:**
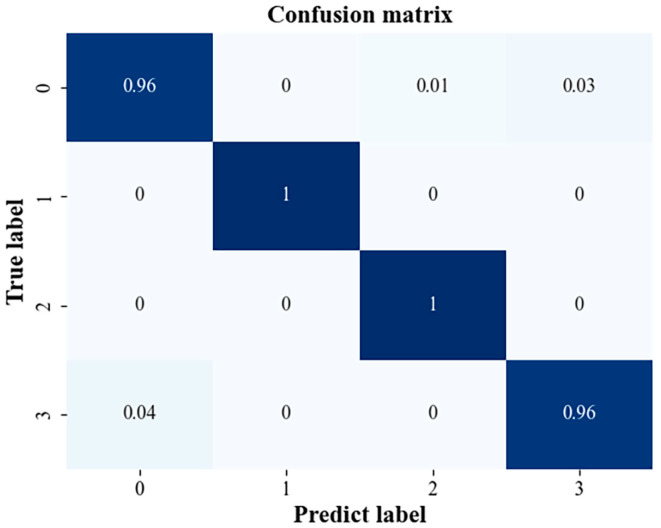
Confusion matrix of sample test results.(The darker the color, the higher the accuracy).

We identified and classified gearbox fault types by SVM: SVM penalty parameter c = 0.75 and kernel function parameter g = 0.14. The confusion matrix of the test sample classification results of this run is shown in Figure 14.

From the sample test results shown in Figure 14, it can be seen that the recognition rate of label 1 (broken gear tooth) and label 2 (gear wear) reached 100% in this recognition process. It shows that the two states of gear broken tooth and gear wear can be identified very accurately. For label 0 (gear pitting) and label 3 (gear normal), the recognition rate was relatively low, reaching 96%. For label 0 (gear pitting) and label 3 (gear normal), the recognition rate was relatively low, reaching 96%. Some 1% of gear pitting conditions were mistaken for worn gear conditions and 3% for normal conditions, while a reading of 4% of the normal state of the gear was mistaken for the pitting state of the gear. Overall, the recognition accuracy of this model is high, which confirms its feasibility for gearbox fault diagnosis.

**Figure 15:**
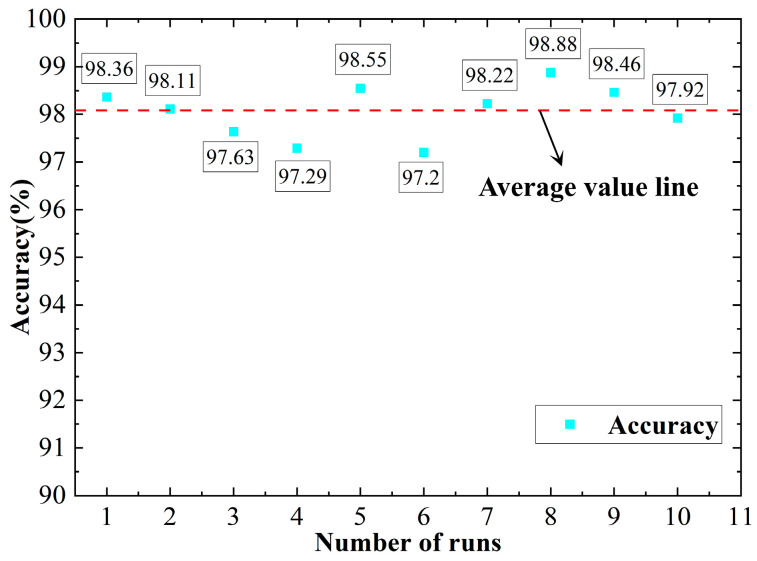
Multidomain information fusion CNN network model recognition rate from 10 runs.

To prevent contingencies in the results, the program was run multiple times. The results from running the program 10 times are shown in Figure 15.

Figure 15 presents the recognition accuracy results from 10 runs of the multidomain information fusion CNN network model. Of these, the fifth run had the highest accuracy, reaching 98.88%. The sixth run had the lowest recognition rate at 97.20%. The results from the 10 runs basically fluctuated around 98.08%. The average accuracy rate was 98.08%. The overall fluctuation was not large, and the stability was good.

### 5.6. Comparative Analysis

#### 5.6.1. Comparative Analysis of Other Methods

Finally, in order to further verify the superiority of the multidomain information fusion CNN network model, we used the FFT-2DCNN, 1DCNN-SVM and 2DCNN-SVM models for comparison and verification. The average value was obtained for each run 10 times, and the final diagnostic results and standard deviations are shown in Table 4.

Under the same experimental conditions, four fault diagnosis methods were compared. The FFT-2DCNN model performs FFT on the original vibration signal of the gearbox to obtain a grayscale image. Then, the grayscale data set obtained by FFT is sent to 2DCNN for fault identification and classification. The 1DCNN-SVM model directly puts the original vibration signal of the gearbox into a one-dimensional convolutional neural network for feature extraction. Then, it uses the support vector machine to identify and classify the faults. The 2DCNN-SVM model puts the original vibration signal of the gearbox into a two-dimensional convolutional neural network for feature extraction and then the support vector machine is used in place of the Softmax layer for fault recognition and classification. The selection of experimental samples for all methods was carried out in the same way as in the experimental design parameters of this study. As can be seen from Table 3, by comparing the accuracy of the four fault diagnosis methods, the fault recognition accuracy of the multidomain information fusion CNN model proposed in this paper reaches 98.08%. Compared with FFT-2DCNN, 1DCNN-SVM and 2DCNN-SVM, our method increases the accuracy rates by 4.46%, 7.43% and 3.56%, respectively. The comparison of the diagnosis results of the various fault diagnosis methods shows that the model proposed in this paper has the highest test accuracy and lowest standard deviation. The lifting effect has obvious advantages and is more suitable for application to gearbox fault diagnosis and identification.

#### 5.6.2. Comparative Analysis of Standard Data Sets

In order to further prove the superiority and stability of the model. This paper uses the Case Western Reserve University data set for analysis. The experiment uses a 1.5 kW three-phase motor, a torque sensor and a dynamometer. The model of the bearing to be tested is SKF6205-2RSJEM deep groove ball bearing, and the sampling frequency is 48000 Hz. The experimental data set is shown in Table 5.

It can be obtained from Table 5 that the data contain 10 fault categories. The fault diameters of the inner ring, rolling element and outer ring are, respectively set to 0.007 inches, 0.014 inches and 0.021 inches. The normal state acts as a special fault type. There are 1000 sets of faults for each type, and a total of 10,000 sets of samples. The data set is divided according to a ratio of 7:2:1.

Under the same experimental conditions, compare the four fault diagnosis methods mentioned in 5.6.1. The average accuracy and standard deviation are shown in Table 6.

It can be seen from Table 6 that the multidomain information fusion CNN model proposed in this paper has a fault recognition accuracy rate of 99.28%. Compared with FFT-2DCNN, 1DCNN-SVM, and 2DCNN-SVM, the accuracy rates by 4.04%, 6.91%, and 2.43% higher, respectively. The standard deviation of the model proposed in this paper is the smallest at 0.3579. This also proves again that the model has good test accuracy and stability.

## 6. Conclusions

This paper proposes and verifies a gearbox fault diagnosis method based on a multidomain information fusion CNN model. A JZQ250 fixed-axis gearbox was used to design and build a fault diagnosis experiment, which verified the effectiveness of the method. An acceleration sensor was used to obtain the original vibration signal of the gearbox, and SVD was used for signal preprocessing and noise reduction. Then, the parallel structure was used to simultaneously perform 1DCNN and 2DCNN for feature extraction. Finally, SVM was used for pattern recognition and classification. The conclusions are as follows:

(1) The gearbox fault diagnosis method based on a multidomain information fusion CNN model is feasible and effective. The model combines a 1D gearbox vibration signal, an STFT 2D time–frequency map and a CNN. CNN multifeature fusion is used to enrich the features of two different dimensions, and two-channel random features are pooled and fused into a one-dimensional feature array. The extracted features are fully enhanced and fused to achieve the purpose of intelligent gearbox fault diagnosis. The model also avoids the incomplete expression of feature information caused by feature extraction and the low accuracy of traditional pattern recognition methods.
(2) A comparison of the model proposed in this paper with the FFT-2DCNN, 1DCNN-SVM and 2DCNN-SVM models shows that the method proposed in this paper has higher accuracy and stronger generalization ability. In addition, it provides a new conceptualization of a physical model for gearbox fault diagnosis and identification.
(3) In research on the fault diagnosis of future rail vehicle gearboxes, multiple sensors can be sampled for data acquisition and multiphysics domain data fusion in order to improve the accuracy of the diagnostic results.

## Figures and Tables

**Table 1:** Multidomain Information Fusion Convolutional Neural Network Structural Parameters.

Channel	Network Layer	Convolution Kernel Size @ Step Size	Activation Function
Channel 1	Input	64 × 64	
	Conv2D-1	64 × 64@6	ReLU
	MaxPooling2D-1	32 × 32@6	
	Conv2D-2	32 × 32@8	ReLU
	MaxPooling2D-2	16 × 16@8	
	Conv2D-3	16 × 16@12	ReLU
	MaxPooling2D-3	8 × 8@12	
	Dropout	0.5	
	FC1	1 × 768	
Channel 2	Input	1 × 1024	
	Conv1D-1	1 × 1024@6	ReLU
	MaxPooling1D-1	1 × 512@6	
	Conv1D-2	1 × 512@8	ReLU
	MaxPooling1D-2	1 × 256@8	
	Conv1D-3	1 × 256@12	ReLU
	MaxPooling1D-3	1 × 128@12	
	Dropout	0.5	
	FC2	1 × 1536	
Fusion	Concatenate	1 × 2304	
	FC3	1 × 128	ReLU
	FC4	1 × 4	

**Table 2:** Fault form data.

Type	Gearbox Status	Data Length	Motor Speed/r/min	Number of Data Groups
1	Pitting	1024	900	1000
2	Broken	1024	900	1000
3	Wear	1024	900	1000
4	Normal	1024	900	1000

**Table 3:** Numbers of training, validation and test sets.

Gearbox Status	Number of Training Sets	Number of Validation Sets	Number of Test Sets	Label
Pitting	700	200	100	0
Broken	700	200	100	1
Wear	700	200	100	2
Normal	700	200	100	3
Total number of samples	2800	800	400	

**Table 4:** The average accuracy and standard deviation of the four models.

Diagnosis Method	FFT-2DCNN	1DCNN-SVM	2DCNN-SVM	Multidomain Information Fusion CNN Model
Ten times average classification accuracy/%	93.62	90.65	94.52	98.08
Standard deviation/%	2.2866	2.3798	0.9231	0.5223

**Table 5:** Experimental data set.

Gearbox Status	Fault Diameter/in	Number of Training Sets	Number of Validation Sets	Number of Test Sets
Inner race fault	0.007	700	200	100
	0.014	700	200	100
	0.021	700	200	100
Rolling element failure	0.007	700	200	100
	0.014	700	200	100
	0.021	700	200	100
Outer race fault	0.007	700	200	100
	0.014	700	200	100
	0.021	700	200	100
Normal	normal	700	200	100

**Table 6:** Accuracy and Standard Deviation.

Diagnosis Method	FFT-2DCNN	1DCNN-SVM	2DCNN-SVM	Multidomain Information Fusion CNN Model
Ten times average classification accuracy/%	95.24	92.37	96.85	99.28
Standard deviation/%	1.7625	1.9236	0.8264	0.3579

## Data Availability

The data used to support the finding of this study are available from the corresponding author upon request.
